# CcpA and CodY Regulate CRISPR-Cas System of *Streptococcus mutans*

**DOI:** 10.1128/spectrum.01826-23

**Published:** 2023-06-27

**Authors:** Da-Young Kang, Andy Kim, Jeong Nam Kim

**Affiliations:** a Department of Integrated Biological Science, College of Natural Sciences, Pusan National University, Busan, Republic of Korea; b Department of Chemistry and Biochemistry, Texas Tech University, Lubbock, Texas, USA; c Department of Microbiology, College of Natural Sciences, Pusan National University, Busan, Republic of Korea; University of Florida College of Dentistry

**Keywords:** *Streptococcus mutans*, CRISPR-Cas system, CcpA, CodY, (p)ppGpp

## Abstract

Clustered regularly interspaced short palindromic repeats (CRISPR) and CRISPR-associated (Cas) genes are widely recognized as bacterial adaptive immune systems against invading viruses and bacteriophages. The oral pathogen Streptococcus mutans encodes two CRISPR-Cas loci (CRISPR1-Cas and CRISPR2-Cas), and their expression under environmental conditions is still under investigation. In this study, we investigated the transcriptional regulation of *cas* operons by CcpA and CodY, two global regulators that contribute to carbohydrate and (p)ppGpp metabolism. The possible promoter regions for *cas* operons and the binding sites for CcpA and CodY in the promoter regions of both CRISPR-Cas loci were predicted using computational algorithms. We found that CcpA could directly bind to the upstream region of both *cas* operons, and detected an allosteric interaction of CodY within the same region. The binding sequences of the two regulators were identified through footprinting analysis. Our results showed that the promoter activity of CRISPR1-Cas was enhanced under fructose-rich conditions, while deletion of the *ccpA* gene led to reduced activity of the CRISPR2-Cas promoter under the same conditions. Additionally, deletion of the CRISPR systems resulted in a significant decrease in fructose uptake ability compared to the parental strain. Interestingly, the accumulation of guanosine tetraphosphate (ppGpp) was reduced in the presence of mupirocin, which induces a stringent response, in the CRISPR1-Cas-deleted (ΔCR1*cas*) and both CRISPR-Cas-deleted (ΔCRD*cas*) mutant strains. Furthermore, the promoter activity of both CRISPRs was enhanced in response to oxidative or membrane stress, while the CRISPR1 promoter activity was reduced under low-pH conditions. Collectively, our findings demonstrate that the transcription of the CRISPR-Cas system is directly regulated by the binding of CcpA and CodY. These regulatory actions play a crucial role in modulating glycolytic processes and exerting effective CRISPR-mediated immunity in response to nutrient availability and environmental cues.

**IMPORTANCE** An effective immune system has evolved not only in eukaryotic organisms but also in microorganisms, enabling them to rapidly detect and neutralize foreign invaders in the environment. Specifically, the CRISPR-Cas system in bacterial cells is established through a complex and sophisticated regulatory mechanism involving specific factors. In this study, we demonstrate that the expression of two CRISPR systems in S. mutans can be controlled by two global regulators, CcpA and CodY, which play critical roles in carbohydrate metabolism and amino acid biosynthesis. Importantly, our results show that the expression of the CRISPR-Cas system in S. mutans influences (p)ppGpp production during the stringent response, which is a gene expression regulatory response that aids in environmental stress adaptation. This transcriptional regulation by these regulators enables a CRISPR-mediated immune response in a host environment with limited availability of carbon sources or amino acids, while ensuring efficient carbon flux and energy expenditure to support multiple metabolic processes.

## INTRODUCTION

Microorganisms are frequently exposed to the risk of invasion by mobile genetic elements in their environment (1). Because these elements are not self-replicable, they must invade the appropriate host for replication, leading to horizontal gene transfer (2). The acquisition of new genetic materials positively affects the genome evolution of the host; however, some events can also disrupt central cellular processes, leading to cell death (2). Bacteria and archaea have evolved defense strategies to cope with the invasion of genetic elements encountered in the environment. Among these, the clustered regularly interspaced short palindromic repeat (CRISPR)-associated (Cas) system is a bacterial adaptive immune system that memorizes partial DNA flanked by repeated sequences in its genome (3). Since the discovery of a repetitive stretch of DNA in Escherichia coli K-12, the CRISPR-Cas system has been identified in 39% of bacterial genomes and 88% of archaeal genomes (4).

In E. coli K-12, the CRISPR-Cas system is activated during growth in medium supplemented with glucose (5, 6). The cAMP receptor protein (CRP) regulates genes involved in carbohydrate utilization and is activated under low-glucose conditions (7). Yang et al. demonstrated that the activation of CRP by glucose depletion inhibits transcription of the *cas* operon (6). In addition, the CRISPR-Cas system of Salmonella enterica serovar Typhi is activated by leucine-responsive protein (LRP) when grown in an N-minimal medium lacking magnesium and phosphate, where the expression of genes involved in survival and pathogenicity increases (8). These results suggest a connection between CRISPR-Cas system expression and pathogenesis in Salmonella strains (8). Thus, the control of the CRISPR-Cas system by these regulators provides new insights into the response of this system to nutrient conditions, in addition to bacterial immunity.

Streptococcus mutans, a causative agent of caries, encodes two CRISPR-Cas systems in its genome: type II-A and type I-C systems (designated CRISPR1-Cas and CRISPR2-Cas, respectively) (9–11). Because Cas1 (encoded by SMU.1757c and SMU.1754c) and Csd1 (SMU.1762c) in the CRISPR2-Cas system of S. mutans strains appear to be truncated, CRISPR2 has been hypothesized to lose its ability to incorporate a novel spacer into the spacer array (9, 12). Moreover, the CRISPR1-Cas (type II-A) system can prevent the transformation of highly matched sequences with protospacers, whereas the CRISPR2-Cas system does not respond (9, 13). Although the functional activity of CRISPR2 in S. mutans immunity remains unclear, the CRISPR1-Cas system is responsible for defense mechanisms against invading genetic material (9).

Both S. mutans CRISPRs are involved in stress tolerance to temperature, and the expression of the CRISPR1-Cas system can be enhanced under oxidative stress or DNA-damaging conditions (13). In particular, a transcriptome analysis of S. mutans performed by another group has shown that the expression of *cas* genes in both CRISPRs is reduced in strains lacking catabolite control protein A (CcpA) or CodY transcriptional regulators for carbohydrate metabolism and amino acid biosynthesis, respectively (14, 15). In S. mutans, CcpA and CodY are primary global regulators that optimize growth by regulating gene expression, which is implicated in carbohydrate metabolism and branched-chain amino acid (BCAA; isoleucine, leucine, and valine) biosynthesis, respectively (14, 15). CcpA activates a regulatory mechanism known as carbon catabolite repression (CCR), which inhibits the expression of genes required to utilize secondary carbon sources in the presence of preferred carbohydrates (14, 16). The histidine-containing protein (HPr) of the phosphoenolpyruvate (PEP)-dependent phosphotransferase system (PTS) mediates CCR by interacting with CcpA to efficiently utilize a carbon source (17). Phosphorylated HPr residues are the key to CcpA activity. When the PEP level is high, the histidine residues of HPr are phosphorylated to take in sugars via PTS activity (17). Under CCR conditions, the intracellular concentration of glycolysis intermediates (fructose-1,6-bisphosphate or glucose-6-phosphate) is elevated, and the 46th serine residue of Hpr is phosphorylated (18). Serine-phosphorylated HPr forms a complex with CcpA and promotes DNA binding of CcpA to the conserved nucleotide sequence, the so-called *cre* (catabolite responsive elements), resulting in the activation or repression of gene expression under CCR control. Two conserved *cre* sites have been identified in S. mutans: *cre1*, WWDWAARCGTTTWMWW (19) and *cre2*, TTTTYHWDHHWWTTTY (20).

CodY was first discovered as a repressor in Bacillus subtilis and is conserved in Gram-positive bacteria with low G+C content (21). CodY binds to a conserved sequence, namely, the CodY box (AATTTTCNGAAAATT), and regulates the expression of genes to allow cells to adapt to low-nutrient conditions (22). In S. mutans, the genes involved in amino acid biosynthesis are repressed by CodY in the presence of BCAAs as effectors of allosteric interactions (15, 23, 24). When S. mutans faces nutrient limitation, the molecular alarmone (p)ppGpp (nutritional alarmones, guanosine pentaphosphate, or tetraphosphate) is synthesized from GDP or GTP, and the accumulation of (p)ppGpp allows bacteria to decrease the expression of genes involved in macromolecular synthesis and increase the expression of genes responsible for amino acid biosynthesis or stress tolerance (21, 25, 26). This phenomenon mediated by (p)ppGpp is known as the “stringent response” and is extensively distributed in bacteria as an adaptation system (25, 27). Because a basal level of (p)ppGpp is required for optimal growth of S. mutans, a strain that does not produce (p)ppGpp cannot grow properly in valine- and leucine-deficient minimal media because of the repressor activity of CodY (15). These data indicate a relationship between the intracellular concentration of (p)ppGpp and the repressor activity of CodY in the expression of gene(s) for stringent responses (15).

In this study, a high sequence similarity between two *cre* sites and a CodY box was found within the promoter region of CRISPR-Cas systems in S. mutans, and we investigated whether *the* S. mutans CRISPR-Cas system could be directly bound to two regulators, CcpA and CodY. Furthermore, we assessed how these systems are integrated into carbohydrate or (p)ppGpp metabolism through transcriptional regulation. Using molecular techniques, we identified the binding sites for CcpA and CodY in the promoter regions of both CRISPRs, and their interaction with CcpA and CodY led to the negative regulation of *cas* gene expression. Notably, deletion of the *cas* operon resulted in reduced fructose intake and low (p)ppGpp accumulation through RelA activity. These results indicate that transcription of the CRISPR-Cas system in S. mutans is connected to fructose-specific PTS activity and (p)ppGpp production induced by RelA synthetase. Thus, understanding the contribution of the CRISPR-Cas system to S. mutans physiology will provide new insights into its integration into cellular processes and stress responses.

## RESULTS

### Genetic organization of CRISPR loci in *Streptococcus mutans* UA159.

**FIG 1:**
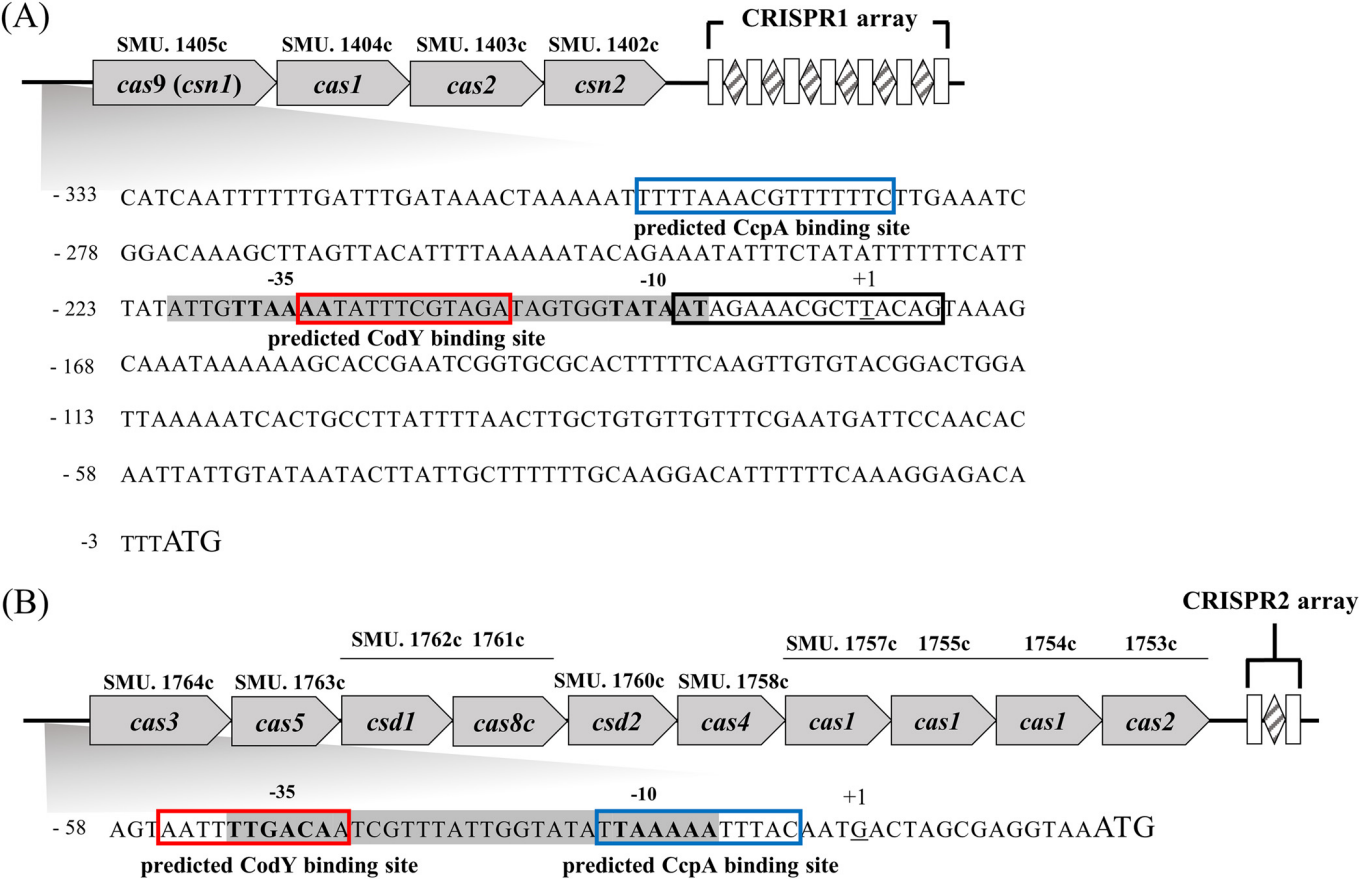
Genetic organization of the CRISPR-Cas systems in S. mutans UA159. Analysis of the upstream regions of the *cas* genes in CRISPR1-Cas (A) and CRISPR2-Cas (B) identified putative promoter regions (shaded in gray) containing the −10 and −35 regions, transcriptional start sites (underlined), and predicted CcpA or CodY binding sequences (blue and red colored boxes, respectively). Consensus sequences for two *cre* sites (*cre1*, WWDWAARCGTTTWMWW; *cre2*, TTTTYHWDHHWWTTTY) and the CodY-binding motif (AATTTTCNGAAAATT) were used to predict the binding sites. The CcpA binding region provided by RegPrecise database (https://regprecise.lbl.gov/) is represented by the black colored box. White boxes and striped diamonds indicate repeats and spacers, respectively, within the CRISPR arrays.

Through the analysis of genome sequences, S. mutans UA159 was found to encode two CRISPR-Cas systems, designated CRISPR1-Cas and CRISPR2-Cas (13). CRISPR1 and CRISPR2-Cas systems are classified as type II-A and type I-C, respectively. Four genes, *cas9* (also named *csn1*; SMU.1405c), *cas1* (SMU.1402c), *cas2* (SMU.1403c), and *csn2* (SMU.1402c), were identified in the CRISPR1-Cas system (Fig. 1A). The CRISPR1 array, following SMU.1402c, contains seven repeat sequences and six spacers (9). Cas9 possesses nuclease activity and is a core protein of the type II-A CRISPR system; *cas1*, *cas2*, and *csn2* are involved in the acquisition of spacers (2, 13). A set of *cas* genes in CRISPR2 was annotated as SMU.1764c through SMU.1753c, and consisted of 10 individual genes (Fig. 1B) (13). Cas3 (SMU.1764c), a signature protein of subtype I-C, acts as a nuclease, and Cas4, Cas1, and Cas2 are involved in spacer acquisition. Moreover, Cas5 (SMU.1763c; also named Cas5d or Cas5c) has catalytic activity as a RNase and exhibits substrate plasticity, with sequence variations at the cleavage site (13, 28). Although the functions of Csd5, Csd1, Cas8c, and Csd2 have not been fully elucidated, these proteins appear to be members of the Cas complex. Notably, Csd2 is highly similar to the Cas proteins, which are involved in CRISPR RNA (crRNA) processing (2, 13). Intriguingly, the CRISPR2 array harbors a 34-bp single spacer flanked by 32-bp repeating sequences at both ends (9).

Using BPROM software (http://linux1.softberry.com/), possible promoters were predicted upstream of each *cas* gene in both CRISPRs. However, all *cas* genes for the individual CRISPRs were in an operon structure under the control of a single promoter (13, 29), as confirmed by reverse transcriptase (RT)-PCR (Fig. S1 in the supplemental material). CRISPR expression can be modulated in strains lacking CcpA or CodY (14, 15). Sequence analysis of *cas* operon using the Clustal Omega program (http://www.ebi.ac.uk/Tools/msa/clustalo/) revealed possible binding regions for CcpA or CodY in the promoter of the *cas* operon in the CRISPR1- and CRISPR2-Cas systems (here referred to as P_CR1_*_cas_* and P_CR2_*_cas_*, respectively) (Fig. 1). In addition, the regulatory interaction between CcpA and the Cas9 promoter in S. mutans CRISPR1-Cas was predicted using the RegPrecise database (https://regprecise.lbl.gov/).

### Binding of CcpA and CodY with a promoter of CRISPR-Cas systems.

**FIG 2:**
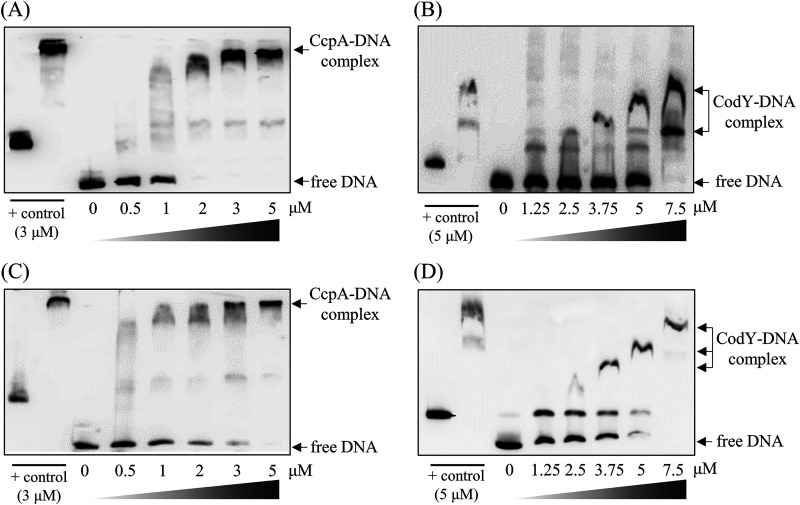
*In vitro* binding of CcpA and CodY to the promoter of the *cas* operons. Biotin-labeled promoter DNA probes (2.5 ng) were incubated with purified CcpA protein (0.5 to 5 μM) or CodY protein (1.25 to 7.5 μM) at 37°C for 30 min. The direct interactions of CcpA with the biotinylated CRISPR1 promoter (A), CodY with the CRISPR1 promoter (B), CcpA with the CRISPR2 promoter (C), and CodY with the CRISPR2 promoter (D) were examined. The *ilvE* or *codY* probes were used as positive (+) controls. For CodY binding to DNA fragments, branched-chain amino acids (BCAAs) were added as effectors at a final concentration of 10 mM. Arrows indicate the migration of either the protein-DNA binding complex or free (unbound) DNA.

To determine whether CcpA or CodY could directly bind to the predicted binding site in the promoter regions of individual CRISPRs, we performed electrophoretic mobility shift assays (EMSAs) (Fig. 2). Recombinant His_6_-tagged CcpA or CodY proteins were purified under native conditions as described in the Methods section. Biotinylated probes of *ilvE* and *codY* were used as positive controls for CcpA or CodY binding, respectively (24). Shifts were detected at concentrations of 2 and 7.5 μM for CcpA and CodY, respectively, indicating that both CcpA and CodY were capable of binding directly to the promoter region of the CRISPR1-Cas system (Fig. 2A and B). Additionally, in the presence of CcpA or CodY, interactions with the CRISPR2-Cas promoter were observed with a band shift in a concentration-dependent manner, suggesting that these proteins can directly bind to the promoter of the CRISPR2-Cas system (Fig. 2C and D). Notably, consistent with recent studies showing that BCAAs are required as effector molecules for the efficient binding of S. mutans CodY protein (23, 30), no shift in CodY with the labeled promoter regions was detected in the absence of BCAAs (Fig. S2).

### Analysis of binding sequences for CcpA and CodY within a CRISPR promoter.

**FIG 3:**
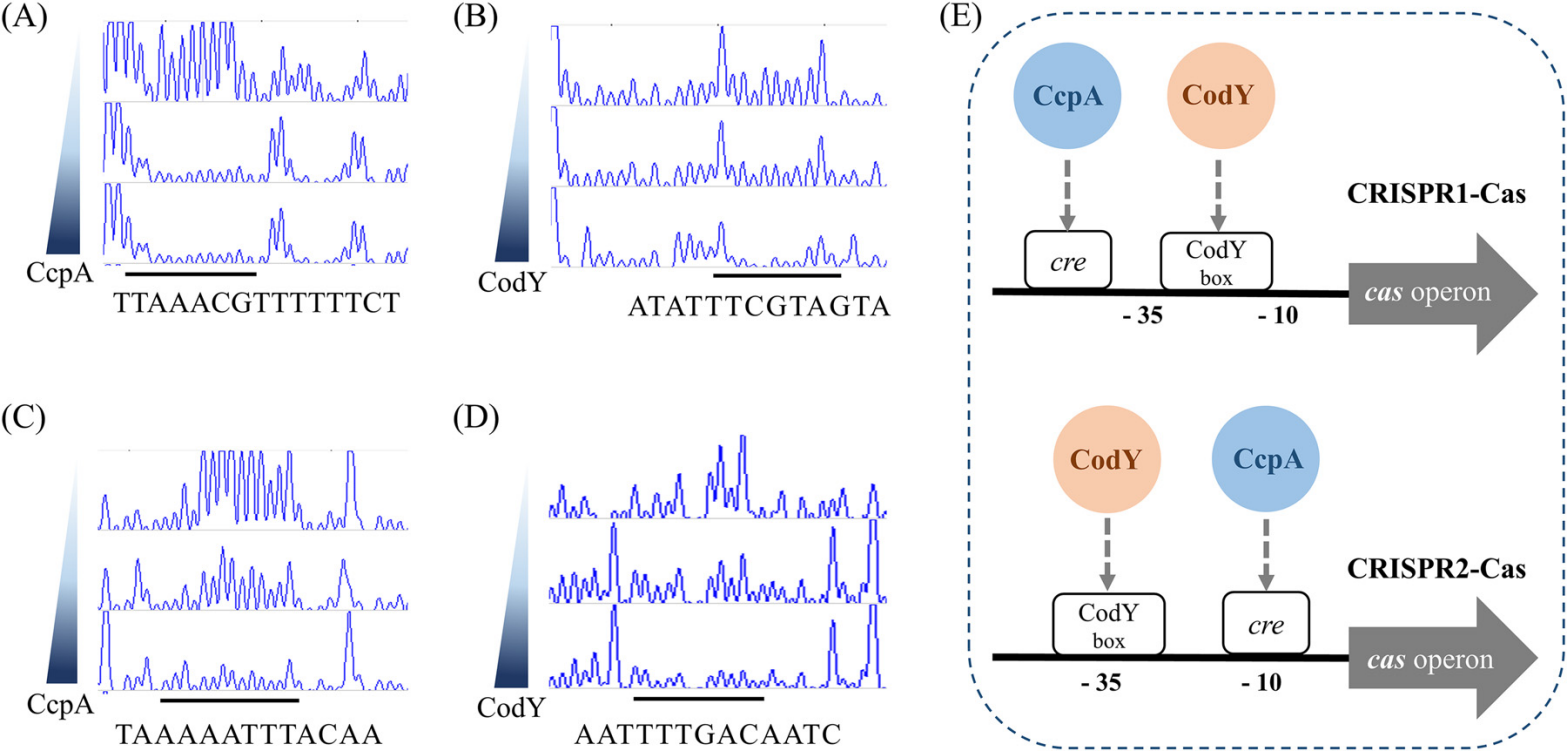
Determination of *cre* site and CodY box. DNase I footprinting assays were performed using biotin-labeled promoter DNA probes (350 ng) to analyze the protected region of CRISPR1-Cas by CcpA (A) or CodY (B), and the region of CRISPR2-Cas by CcpA (C) or CodY (D). The binding site is underlined, and the sequence is indicated. The increasing concentrations (0, 13.66, and 27.31 μM for CcpA; 0, 27.62, and 55.23 μM for CodY) of His-tagged proteins are shown at the top, middle, and bottom of each panel. (E) Based on the electropherograms, the binding regions of CcpA and CodY to the CRISPR1 or CRISPR2 promoter regions are illustrated as shown. Notably, the CodY box and *cre* site on the promoter of CRISPR2-Cas overlap the −10 or −35 element for RNA polymerase.

EMSA results showed that CcpA and CodY could directly bind to the promoter regions of the two CRISPRs. To map the specific binding sites of CcpA and CodY, we performed DNase footprinting assays using non-radiochemical capillary electrophoresis (Fig. 3). Biotinylated DNA probes containing potential binding sites for CcpA or CodY proteins were generated with primers labeled by 6-carboxyfluorescein (6-FAM) and biotin at the 5′ and 3′ ends, respectively, via PCR amplification. Nucleotides protected from DNase activity presented no or lower peak signals compared to the results without the addition of the purified protein. In CRISPR1-Cas, a region protected by CcpA (TTAAACGTTTTTCT) was found in the putative promoter that starts 300-bp upstream of the *cas* operon (Fig. 3A). The region protected by CodY (ATATTTCGTAGTA) overlapped the putative promoter and was located 211-bp upstream of the *cas* operon (Fig. 3B). In CRISPR2-Cas, the sequence of the region protected by CcpA was TAAAAATTTACAA, located 28-bp upstream of the start codon of the first *cas* gene (Fig. 3C). The CodY box was also detected as the sequence AATTTGACAATC, located 55-bp upstream of the start codon of the *cas3* gene (Fig. 3D). The *cre* site and CodY box lie in the −10 and −35 regions of the promoter sequence, respectively (Fig. 3E). These findings indicate that the expression of S. mutans CRISPRs could be controlled by the coordinated regulation of CcpA and CodY.

### Effect of CcpA and CodY in the expression of CRISPR genes.

**FIG 4:**
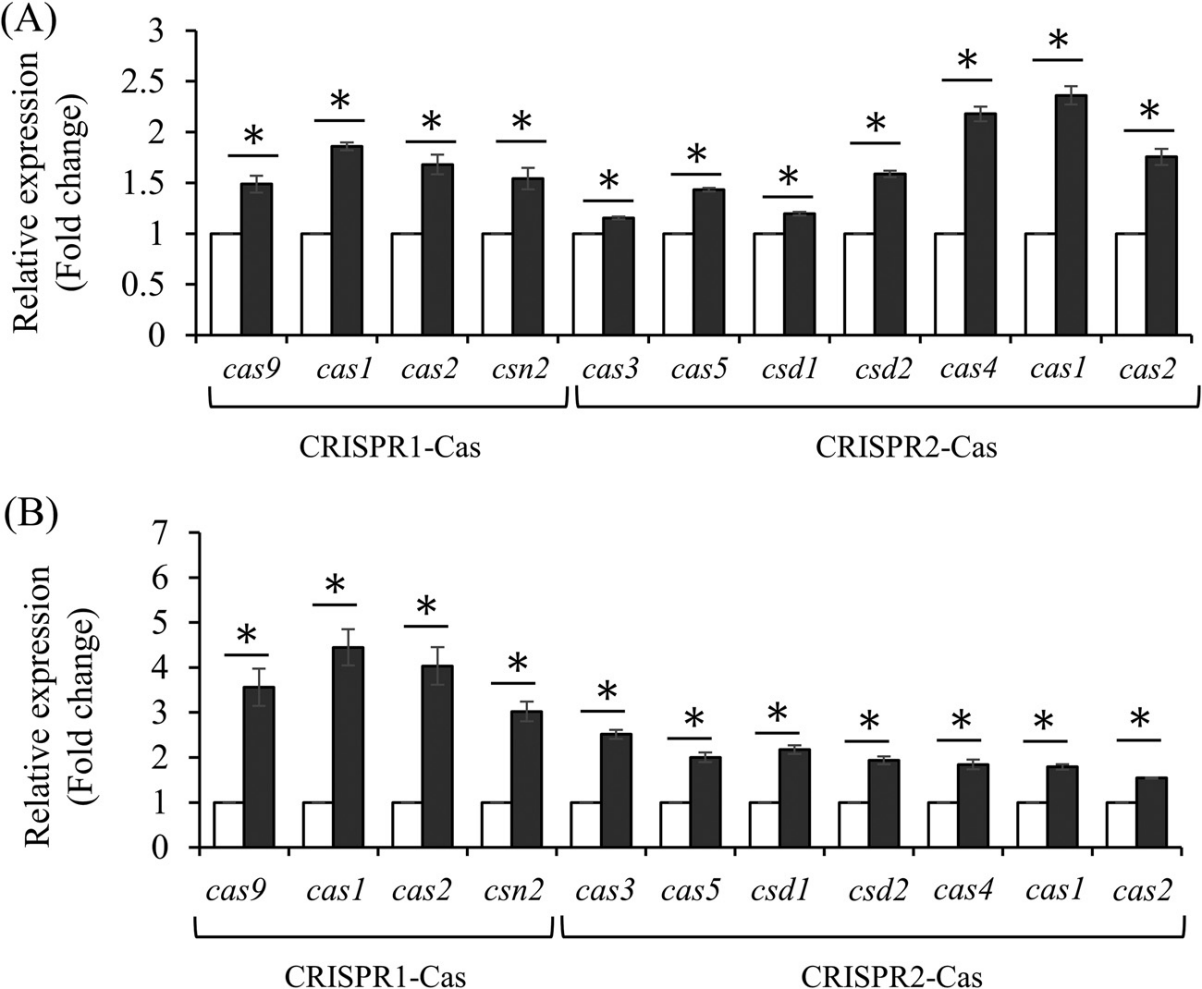
Transcription of genes in *cas* operons. The relative expression level of the *cas* operon was determined in the *ccpA* (A) or *codY* deletion (B) mutants. S. mutans wild-type and mutant strains were grown to an optical density at 600 nm (OD_600_) of 0.7 at 37°C in a 5% CO_2_ atmosphere in BHI medium. The expression of 16S rRNA was used for normalization. All values are the average of three results, and error bars represent the deviation from the mean. Asterisks (*) indicate that data differ from the wild-type genetic background at *P < *0.05 (Student’s *t* test analysis).

To explore whether the direct interaction of CcpA or CodY could modify the transcription of *cas* genes, we compared the expression levels of *cas* genes in Δ*ccpA* or Δ*codY* mutant backgrounds using quantitative real-time PCR (qRT-PCR). The expression of all genes with both CRISPRs was significantly increased in the *ccpA*-deleted background (Fig. 4A). Similarly, a strain lacking *codY* showed enhanced expression of all *cas* genes compared with the parental strain (Fig. 4B). Thus, loss of CcpA or CodY leads to the upregulation of *cas* genes, indicating that CcpA or CodY proteins likely repress the transcription of both CRISPRs by competitive binding of RNA polymerase under certain conditions. Notably, the result showing that transcriptional regulation of CRISPR-associated genes by CcpA and CodY suggests that the S. mutans CRISPR system integrates carbohydrate metabolism and amino acid biosynthesis.

### The expression of CRISPR1-Cas system was enhanced under fructose-rich conditions.

**FIG 5:**
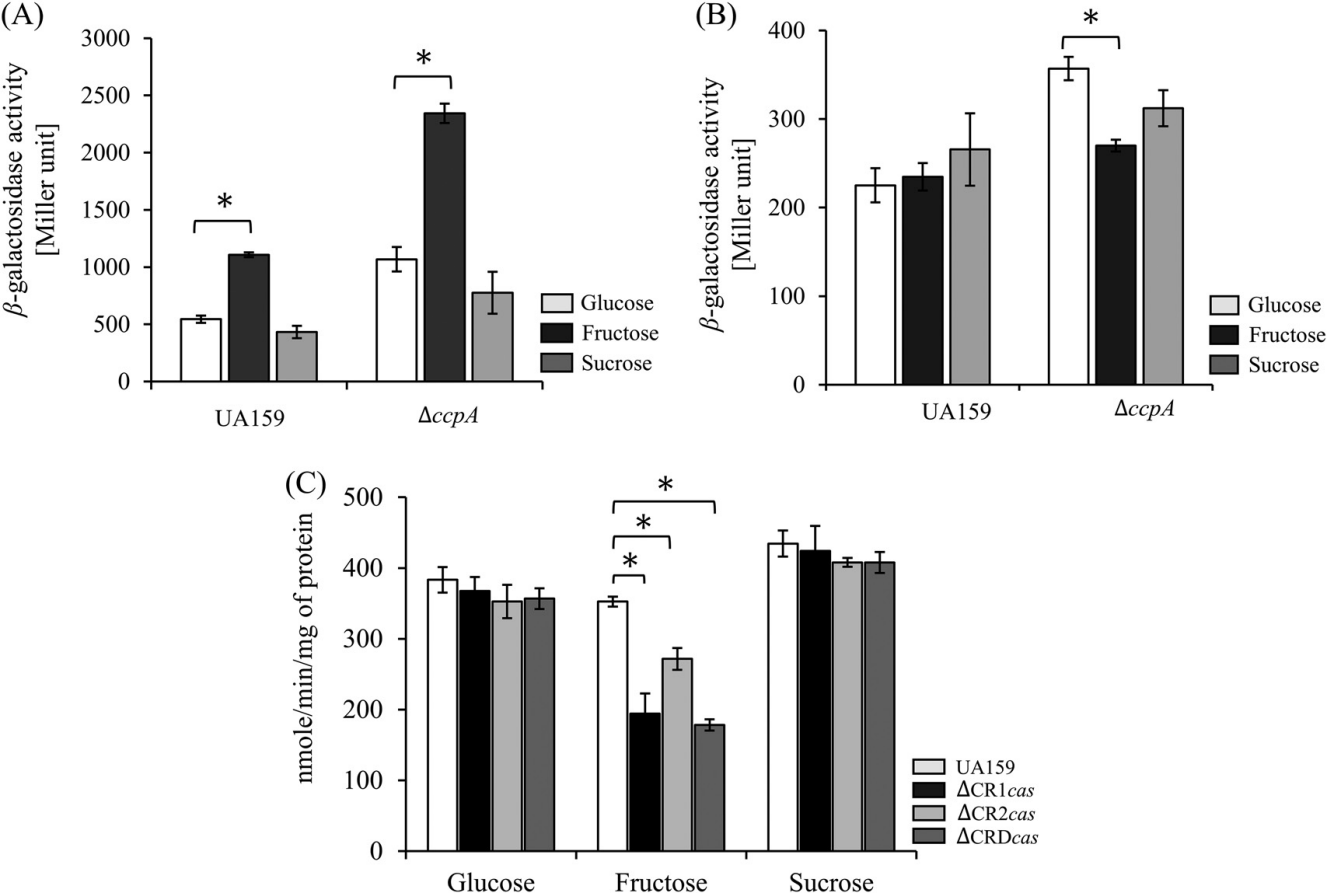
Effects of carbon sources on the expression of the *cas* operons. Cells were cultivated in TV medium containing glucose, fructose, or sucrose at a final concentration of 10 mM and grown to an OD_600_ of 0.5. The activity of the *lacZ*-fused promoter of the CRISPR1-Cas (A) and CRISPR2-Cas (B) was measured in the wild-type and Δ*ccpA* mutant strains as described in Materials and Methods. (C) Sugar transport by the PTS was assayed in the wild-type and CRISPR mutant (single- or double-deletion) strains. All values are the average of three results, and error bars represent the deviation from the mean. Statistical significance was determined by Student’s *t* test; *, *P < *0.05.

Inactivation of the S. mutans *ccpA* gene results in the upregulation of nine genes that encode sugar-specific PTS system components by up to 2-fold when the cells are grown in glucose-containing tryptone-vitamin vase (TV) medium (14). Under the same conditions, the expression of 19 genes encoding hypothetical proteins, including *cas* genes in CRISPR1 (SMU.1404c, SMU.1403c, and SMU.1402c), increased (14). As described above, the promoter region of *cas* operons in S. mutans contains a *cre* site that facilitates CcpA binding. To investigate whether the expression of *cas* operons could be influenced by different carbon sources, we determined P_CR1_*_cas_* and P_CR2_*_cas_* activities in the wild-type and Δ*ccpA* mutant strains using cultures grown in medium supplemented with glucose, fructose, or sucrose. In the wild-type strain, the activity of P_CR1_*_cas_* appeared to increase by 1.92 ± 0.1-fold in a medium containing fructose, compared with glucose conditions (*P < *0.05) (Fig. 5A). Moreover, the promoter activities in response to three carbohydrates were also observed in the mutant lacking *ccpA*; specifically, the P_CR1_*_cas_* activity enhanced to 2.39 ± 0.22-fold under the fructose-rich conditions compared with that under glucose-rich conditions (*P < *0.05). Meanwhile, the activity of P_CR2_*_cas_* in the wild-type and mutant strains showed no significant difference in medium supplemented with the individual sugars. However, the *ccpA* mutant exhibited higher P_CR2_*_cas_* activity, an increase of 1.32 ± 0.65-fold, under glucose conditions (*P < *0.05) (Fig. 5B). Thus, the promoter activity of each CRISPR was differently induced by different carbohydrates, and the introduction of a CcpA deletion further influenced the promoter activities.

Furthermore, to assess whether CRISPR deletion influences the activity of PEP-dependent PTS, we constructed single- or double-deletion mutant strains lacking a single or both *cas* operons (ΔCR1*cas*, ΔCR2*cas*, and ΔCRD*cas*). While no difference in PTS activity was observed between the wild-type and mutant strains under the conditions with glucose or sucrose supplementation, all of the mutant strains exhibited reductions in activity to 2.02 ± 0.18, 1.36 ± 0.06, and 2.04 ± 0.09-folds, respectively, when supplemented with fructose as the sole substrate (*P < *0.05) (Fig. 5C). However, no growth change was observed under any carbohydrate conditions (data not shown).

### Measurement of (p)ppGpp accumulation in CRISPR-Cas mutants.

**FIG 6:**
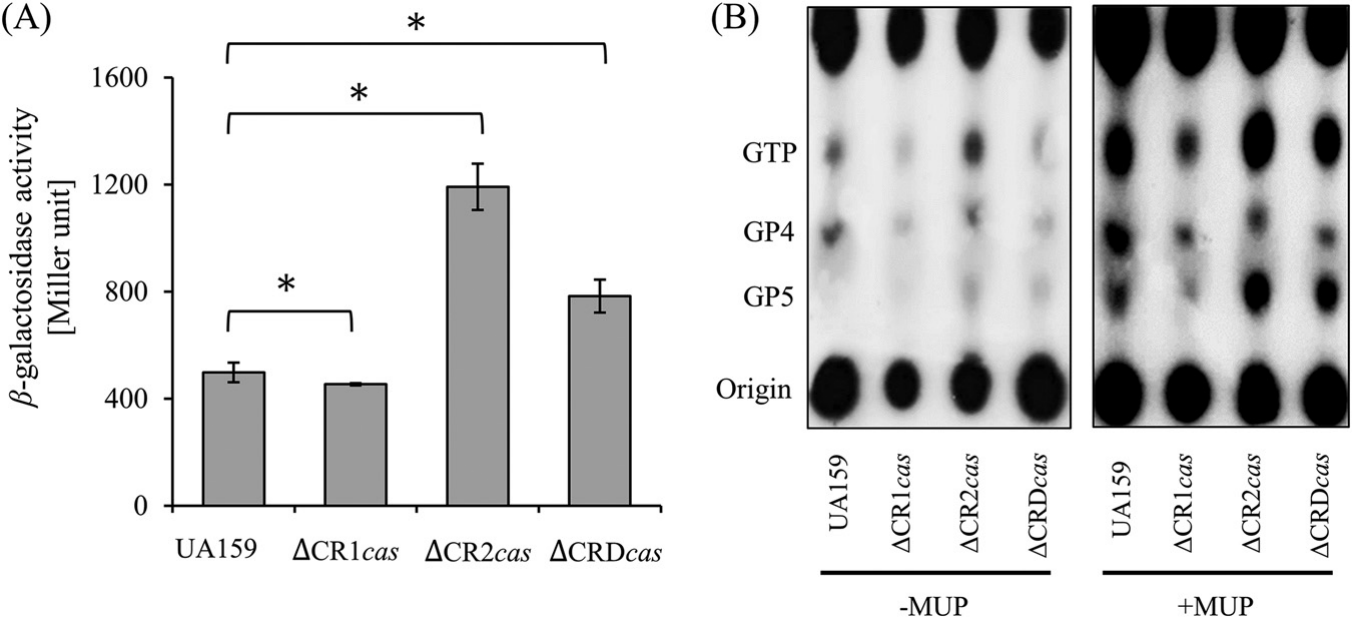
(A) Promoter-*lacZ* fusion activity from the promoter region of *relA*, encoding (p)ppGpp synthetase. Cells were grown to an OD_600_ of 0.5 in defined FMC medium containing 25 mM glucose, and a β-galactosidase assay was performed as described in Materials and Methods. All values are the average of three results and error bars represent the deviation from the mean. Asterisks (*) indicate that data differ from the wild-type genetic background, *P < *0.05. (B) The accumulation of (p)ppGpp in *cas* operon deletion strains. Cells were grown in FMC medium containing 25 mM glucose to an OD_600_ of 0.2 and labeled with [^32^P]orthophosphate for an additional 1 h, with or without 500 ng/mL mupirocin to induce (p)ppGpp synthesis. Each acid extract was spotted on PEI-cellulose plates separated for thin-layer chromatography. The identity of the migrating nucleotides is shown on the left. Images are representative of three independent replicates.

CodY regulates the expression of genes involved in rapid adaptation and persistence under nutrient-deprived conditions (23, 31). In addition, RelA is a major (p)ppGpp synthase in S. mutans that synthesizes most of the (p)ppGpp accumulated during the stringent response (27, 32). Although the relationship between CodY and (p)ppGpp has not yet been elucidated, the regulatory protein, CodY, modulates the expression of genes involved in BCAA biosynthesis if sufficient pools of BCAAs are available under oxygenated conditions (15, 33, 34). This suggests that CodY regulation may be included in the algorithm of global metabolism that occurs during stringent responses. We investigated whether the CRISPR-Cas systems regulated by CodY were associated with a stringent response through the relationship between the accumulation of (p)ppGpp and the CRISPR-Cas systems. To determine the relationship between RelA synthetase and CRISPRs, we introduced a *lacZ*-fused *relA* promoter into the CRISPR deletion mutant background. The activity of P*_relA_* somewhat decreased, 1.11 ± 0.07-fold, in ΔCR1*cas* (*P < *0.05), whereas it increased to 2.49 ± 0.23 and 1.21 ± 0.5-folds in ΔCR2*cas* and ΔCRD*cas*, respectively (*P < *0.05) (Fig. 6A). In a slight contrast, it was observed that the promoter activity of *relP* and *relQ* genes was greatly enhanced only in the double-deletion mutant (data not shown). Next, we tested whether the level of promoter activity correlated with (p)ppGpp accumulation. When mupirocin was added, the accumulation of (p)ppGpp in the ΔCR2*cas* and ΔCRD*cas* strains was relatively higher than that in the wild type, whereas the ΔCR1*cas* strain exhibited lower (p)ppGpp accumulation (Fig. 6B). Here, we found that, similar to the results for the promoter activity of RelA, these CRISPR-Cas systems are required to fine-tune the levels of (p)ppGpp produced by RelA synthetase activity, suggesting interdigitation of the CRISPR-Cas systems into (p)ppGpp metabolism during the stringent response.

### Promoter activity of the CRISPR-Cas systems in various conditions.

**FIG 7:**
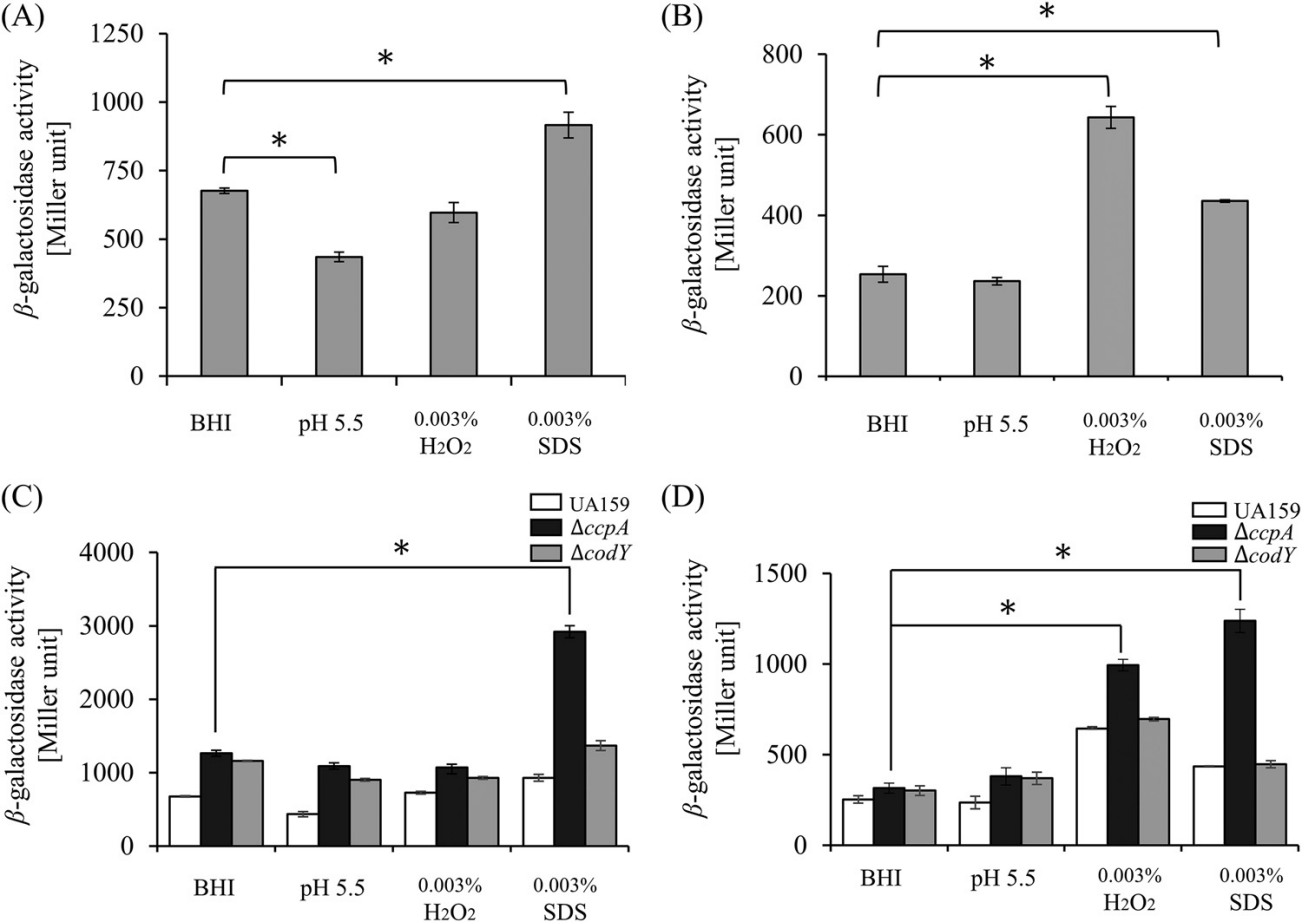
Promoter activity of *cas* operons in various growth conditions. Cells were grown at 37°C in a 5% CO_2_ atmosphere in BHI medium supplemented with HCl to pH 5.5, 0.003% H_2_O_2_, and 0.003% SDS until reaching an OD_600_ of 0.5. The activities of P_CR1_*_cas_* (A) or P_CR2_*_cas_* (B) in the wild-type strain were measured as described in Materials and Methods. Changes in P_CR1_*_cas_* (C) or P_CR2_*_cas_* (D) activity in the Δ*ccpA* and Δ*codY* mutant backgrounds were examined during growth under the same conditions described above. All values are the average of three results, and error bars represent the deviation from the mean. Asterisks (*) indicate that data differ from the cells grown in BHI medium at *P < *0.05 (Student’s *t* test analysis).

A previous study on CRISPR-Cas systems in S. mutans demonstrated that the *cas* operon of the CRISPR1-Cas system is mainly involved in the response to environmental stressors and that both systems have a synergistic role in sensitivity to high temperatures (13). To investigate whether the expression of *cas* operons could be modified by the stress conditions tested, we measured the promoter activity of *cas* operons. Hydrogen peroxide released by commensal bacteria such as Streptococcus sanguinis and Streptococcus gordonii is an oxidative stressor for S. mutans (35), which can be converted into hydroxyl radicals, damaging the synthesis of macromolecules such as proteins and DNA (36). Sodium dodecyl sulfate (SDS) is a detergent that penetrates the cell membrane, creatin a membrane stress (13). The activity of P_CR1_*_cas_* increased 1.35 ± 0.02-fold (*P < *0.05) in 0.003% SDS, decreased 1.56 ± 0.01-fold at pH 5.5, and decreased 1.14 ± 0.02-fold in 0.003% H_2_O_2_ (Fig. 7A). The activities of P_CR2*cas*_ in 0.003% H_2_O_2_ and 0.003% SDS increased 2.67 ± 0.33-fold (*P < *0.05) and 1.73 ± 0.18-fold (*P < *0.05), respectively, suggesting the CRISPR2-cas played a role in responding to extracellular oxidative stress (H_2_O_2_). In general, low-acid conditions are generated by catabolism of S. mutans, which produces organic acids from a wide range of carbohydrate conversions (37). An increase in available carbon sources allows bacteria to perform rapid glycolysis, lowering the internal pH to 4 (35, 37). However, as shown here, weakly acidic conditions (pH 5.5) did not significantly affect the activity of P_CR2_*_cas_* (Fig. 7B).

To assess the expression of CRISPRs under the control of CcpA or CodY in response to environmental stressors, we measured the activities of P_CR1_*_cas_* and P_CR2_*_cas_* under stress conditions in Δ*ccpA* or Δ*codY* backgrounds and compared to that of the wild-type strain. With respect to P_CR1_*_cas_* activity, a similar change to that in the wild-type strain was observed in Δ*codY* under acidic and oxidative stress conditions. Interestingly, the activity of P_CR1_*_cas_* in the Δ*ccpA* background markedly enhanced 2.88 ± 0.09-fold (*P < *0.05) when the cells were treated with SDS to generate cell membrane stress (Fig. 7C). Furthermore, increased P_CR2_*_cas_* activity in both mutant backgrounds was clearly observed in response to H_2_O_2_ or SDS (Fig. 7D). However, the overall changes in both promoter activities appeared to be the result of responses to the environmental stressors tested, not due to CodY or CcpA mutations. Therefore, these results revealed that the two CRISPR systems in S. mutans work cooperatively or singularly depending on the type of environmental stressor (13).

## DISCUSSION

For microorganisms living in rapidly changing environments, the utilization of sufficient carbon sources and the ability to protect against external risks are essential factors (38, 39). Bacteria have adopted various strategies to block external risk factors and control internal metabolism to overcome undesirable conditions (38, 39). Among these strategies, bacteria have evolved an immune mechanism called the CRISPR-Cas system to defend themselves against external genetic material (3). The present study demonstrated transcriptional regulation of CRISPR-Cas systems in S. mutans by allosteric interaction with two global regulators, CcpA and CodY, indicating that *cas* genes belong to a group of genes that are associated with bacterial adaptation to environmental changes. We also propose that the changes in biochemical properties caused by CRISPR deletion result from modifications to carbon and energy fluxes. In particular, our results revealed that the CRISPR-Cas system in S. mutans contributes to cellular processes and persistence, especially fructose intake and the stringent response mediated by (p)ppGpp metabolism.

**FIG 8:**
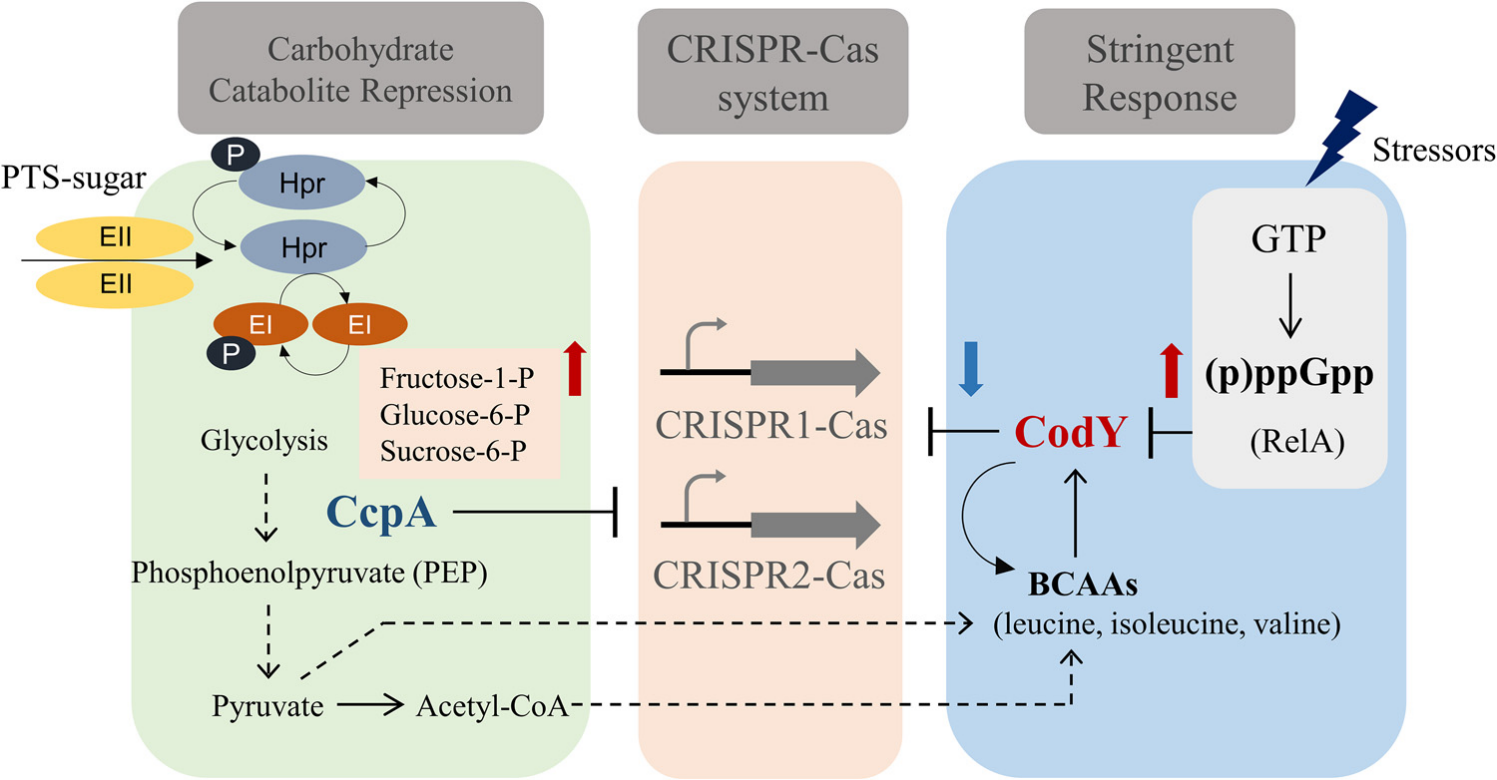
Proposed model illustrating the regulation of CRISPR-Cas systems by CcpA and CodY. CcpA directly binds to the promoter region of both CRISPR-Cas operons, while CodY interacts allosterically with the same regions in the presence of BCAAs. Repression by CcpA can be modulated under different carbohydrate conditions, particularly under fructose-derived CCR conditions. Conversely, CodY repression of CRISPR systems is strongest at the basal level of (p)ppGpp. During the stringent response, CodY partially alleviates this repression through high levels of intracellular (p)ppGpp production by RelA activity, resulting in the upregulation of the CRISRP-Cas system. Red arrows indicate the accumulation of glycolytic intermediates and (p)ppGpp. Blue arrow represents the reduced repression by CodY on CRISPR systems.

Carbohydrates are essential for S. mutans growth because S. mutans lacks a complete respiratory system and cannot perform oxidative phosphorylation (14). A previous study on the CcpA regulon in S. mutans showed that nine genes encoding sugar-specific PTS system components were upregulated 2-fold under TV-glucose conditions (catabolite-repressing conditions) as a result of *ccpA* deletion (14). Under the same conditions, the expression of 19 genes encoding hypothetical proteins, including *cas* genes consisting of CRISPR1-Cas (SMU.1404c, SMU.1403c, and SMU.1402c), increased (14). In S. mutans, growth on fructose can trigger CCR *via* the CcpA pathway (14); however, CCR is primarily induced by the PTS system, which transports sugars from the extracellular environment (18, 33). Based on our understanding of the results and existing knowledge, we propose a model that elucidates the association between CRISPR regulation by CcpA and CodY and carbohydrate and (p)ppGpp metabolism. Our model suggests that the binding affinity of these regulators could be modulated by the levels of metabolic intermediates and effectors (Fig. 8). The promoter activity of both CRISPRs was increased in the Δ*ccpA* strain in all carbohydrates tested compared with the wild-type background, particularly showing high promoter activity of CRISPR1-Cas in the TV-fructose medium. These results partially support our model, indicating that the *cas* operon in CRISPR1-Cas is regulated by CcpA in association with CCR, while also suggesting that other factors may influence this regulation depending on the specific carbohydrate that triggers CCR. Additionally, low PTS activity was observed in all CRISPR deletion mutants under fructose-rich conditions. This regulation may be important for balancing the energy expenditure of the CRISPR-Cas system, which requires a significant amount of energy and can be costly for bacteria. Thus, CcpA helps optimize metabolic pathways in response to environmental cues, while maintaining the adaptive immune response provided by the CRISPR-Cas system. Considering the apparent impact of stress-related regulation on CRISPR-Cas genes, it might not be unreasonable to consider the effect of fructose as part of the “stressor” category, as fructose-1-phosphate has been known to trigger autolysis in S. mutans (40). Therefore, CcpA plays a crucial role in coordinating metabolic processes and immune defense mechanisms in response to environmental changes, allowing S. mutans to efficiently utilize available carbohydrates and respond to stress signals.

The stringent response mediated by (p)ppGpp is widely distributed in bacteria as an adaptation strategy for survival under nutrient limitations. The accumulation of (p)ppGpp activates or inhibits the expression of numerous genes, suggesting that the intracellular (p)ppGpp concentration is a critical point that allows bacteria to select the genes required for survival (21). In S. mutans, RelA is a major (p)ppGpp synthase that is activated in the classical stringent response and is a bifunctional enzyme with both synthase and hydrolase activities (32). Based on the finding that CodY can directly bind to the promoter region of the *cas* operons, we investigated the linkage of (p)ppGpp and CRISPR-Cas to determine whether the CRISPR-Cas system is related to the stringent response. Here, the RelA promoter activity was found to respond differently to the introduction of CRISPR1-Cas and CRISPR2-Cas deletions. Unlike what has been seen in the interaction of CodY with the target DNAs in other bacterial species (1, 41–43), binding of CodY to the *cas* promoter is not responsive to GTP and functions only as a repressor. One major finding of our study, consistent with this result, is that a lower level of (p)ppGpp accumulation was detected in the CRISPR1-Cas mutant during the stringent response. Conversely, the loss of CRISPR2-Cas resulted in a slightly higher production of (p)ppGpp. Based on these findings, we propose that the binding affinity of CodY to the *cas* promoter of CRISPR systems is the strongest at the basal level of (p)ppGpp if the BCAA pools reach the threshold (Fig. 8). Meanwhile, during the stringent response, the CodY repression to CRISPR systems is partially alleviated by the high accumulation of (p)ppGpp with the reduction of the GTP pool (44). This upregulation of CRISPR-Cas activity can lead to a stronger immune response against invading genetic elements, which is crucial for bacterial survival under stress conditions. Furthermore, our proposed model, based on the current data, not only suggests a distinct role for the individual CRISPR systems in the activity of RelA protein but also leaves open the possibility of the involvement of the small proteins RelP and RelQ. Additionally, the observation that the effect of CodY on CRISPR is dependent on growth conditions further supports the notion that environmental changes influence intracellular metabolite/energy pools (e.g., BCAAs and GTP), determining the binding behaviors of these regulators of CRISPR-Cas systems.

### Conclusions.

Our findings reveal that the CRISPR-Cas system in S. mutans, serving as bacterial adaptive immunity, is directly influenced by the interactions of CcpA or CodY, which are associated with various cellular processes. The results presented in this study highlight the intricate relationship between the CRISPR-Cas system in S. mutans and its impact on carbon metabolism, stringent response, and stress tolerance. The transcriptional regulation of the CRISPR systems can be modulated by these two regulators under different physiological conditions arising from rapid environmental changes. Although the current study provides limited information about the transcriptional regulation of CRISPR-Cas systems by CcpA and CodY, the profound connection between CRISPR systems and carbohydrate as well as (p)ppGpp metabolism is evident. This regulation likely involves both direct and indirect effects on the expression of CRISPR systems. Deletion of these regulators not only results in increased expression of *cas* operons but also affects metabolic pathways associated with the intake and utilization of carbohydrates, particularly fructose, leading to genetic and physiological changes. The CcpA-mediated CCR is important for carbon flux and energy metabolism, and its involvement in regulating the CRISPR system is apparent. Furthermore, the depletion of available energy sources stimulates the synthesis and accumulation of alarmone (p)ppGpp, directly regulating stress response genes. The loss of CRISPR systems is considered to have an indirect effect on the biosynthesis of (p)ppGpp molecules, particularly in response to nutritional challenges arising from fluctuations in carbon and energy availability. Overall, our findings provide a novel perspective on the integration of CRISPR systems into the mechanistic regulation of multiple processes in S. mutans. The intricate interplay between the CRISPR-Cas system and regulators CcpA and CodY highlights the adaptability of S. mutans to rapidly changing environmental conditions, enabling effective defense and metabolic responses.

## MATERIALS AND METHODS

### Bacterial strains and growth conditions.

All strains and plasmids used in this study are listed in Table S1. Streptococcus mutans wild-type and mutant strains were routinely grown in brain-heart infusion (BHI) medium (BD Biosciences, MD, USA) at 37°C in a 5% CO_2_ atmosphere, unless stated otherwise. An E. coli strain was aerobically grown in Luria-Bertani (LB) medium (BD Biosciences) at 37°C. When needed, antibiotics were added to the medium as follows: erythromycin (10 μg/mL for S. mutans), spectinomycin (1 mg/mL for S. mutans), kanamycin (50 μg/mL for E. coli and 1 mg/mL for S. mutans), and tetracycline (10 μg/mL for S. mutans).

### Strain construction.

S. mutans mutant strains were constructed by PCR ligation mutagenesis (45) using the primers listed in Table S2. Briefly, 5′- and 3′-flanking regions of the target gene were amplified using chromosomal DNA of S. mutans UA159 as PCR templates. The flanking regions were ligated with an antibiotic marker cassette derived from pJL105 using T4 DNA ligase (Enzynomics, Daejeon, South Korea). The resulting products were transformed into S. mutans and the target gene was replaced with an antibiotic cassette via homologous recombination of the flanking regions. All *cas* genes in CRISPR1-Cas and CRISPR2-Cas were replaced with the spectinomycin cassette (designated ΔCR1*cas*) or kanamycin cassette (designated ΔCR2*cas*), respectively. A double-deletion mutant was constructed using the same method (ΔCRD*cas*). For fusion of the CRISPR promoter, the upstream region of each CRISPR locus was amplified with custom primers (Table S2) and cloned into the pMZ-*lacZ* integration vector (46), which carries the *lacZ* gene lacking a promoter and ribosomal binding site. The resulting construct was transformed into S. mutans to establish a promoter-*lacZ* fusion in a single copy of the chromosome by double-crossover homologous recombination, with *mtlA-phnA* genes serving as the integration site.

### Electrophoretic mobility shift assay (EMSA).

Electrophoretic EMSAs were performed as previously described (47). The 385- and 395-bp upstream regions of each *cas* operon were amplified by primers with biotinylated nucleotides at the 5′ end (Table S2). Binding reactions were conducted in a mixture containing 2.5 ng DNA probe, 10 mM HEPES, 50 mM KCl, 5 mM MgCl_2_, 1 mM EDTA, 5 mM dithiothreitol, 1 μg poly (dI-dC), and 10% glycerol in combination with different concentrations (0.5, 1, 2, 3, and 5 μM CcpA; 1.25, 2.5, 3.75, 5, and 7.5 μM CodY) of purified proteins. Branched-chain amino acids (BCAAs; l-isoleucine, l-leucine, and l-valine) were added as effectors to the reaction mixture at a final concentration of 10 mM when desired. The binding reaction was allowed to occur at 37°C for 30 min, then the results were loaded to a 6% non-denaturing polyacrylamide gel in 0.5× Tris-borate-EDTA buffer. The separated DNAs were transferred onto a nitrocellulose membrane using a Trans-Blot SD Semi-Dry Transfer (Bio-Rad, Hercules, CA, USA). Following UV cross-linking, biotinylated DNAs were detected using a Chemiluminescent Nucleic Acid Detection Module kit (Thermo Fisher Scientific, Rockford, IL, USA), according to the supplier’s protocol.

### DNase I footprinting assay.

The 385-bp and 395-bp upstream regions of each *cas* operon in CRISPR1-Cas and CRISPR2-Cas were amplified with 6-FAM labeled forward primer and biotinylated reverse primer. Each labeled DNA fragment (350 ng) was incubated with increasing concentrations of CcpA (0, 13.66, and 27.31 μM) or CodY (0, 27.62, and 55.23 μM) at 37°C for 30 min in 50 μL binding mixture, the same as used in the EMSA reactions. Two μL of the mixture was separated on a 6% non-denaturing gel to ensure the interaction of the labeled DNAs and proteins, and the remaining sample was treated with 0.1 unit of DNase I at 37°C for 4 min. The enzyme was inactivated by adding EDTA to a final concentration of 60 mM and heated at 85°C for 10 min. The DNase-treated samples were purified using a Centri-Sep column (Applied Biosystems, CA, USA). The purified samples were dried in a vacuum centrifuge without heating and resuspended in 10 μL Hi-Di formamide (Applied Biosystems, Warrington, United Kingdom). For size analysis of the DNA fragments, capillary electrophoresis was performed using a 3730xl DNA Analyzer (Nicem, Seoul, South Korea), and electropherograms were analyzed using the GelQuest program (http://www.sequentix.de/gelquest/).

### β-Galactosidase assay.

The β-galactosidase activity was determined as detailed previously (48) with some modifications. Briefly, overnight cultures were grown in BHI or defined FMC containing 25 mM glucose (for the *lacZ* fused promoter of *relA* encoding a (p)ppGpp synthetase) to an optical density at 600 nm (OD_600_) of 0.5 and harvested by centrifugation at 10,000 × *g* for 5 min. The obtained pellet was washed once with 750 μL of Z-buffer (12 mM Na_2_PO_4_, 8 mM NaH_2_PO_4_, 2 mM KCl, and 0.2 mM MgSO_4_ [pH 7.0]) and was resuspended in 650 μL of Z-buffer containing 50 mM β-mercaptoethanol. Moreover, 25 μL of toluene:acetone (1:9; vol/vol) solution was added and vortexed for 2 min to make a permeabilized cell. The cell suspension (250 μL) was mixed with 50 μL of 4 mg/mL ONPG (*O*-nitrophenyl-β-galactoside) solution and incubated at 37°C until the color changed to yellow. Once the yellow color was observed, the reaction was stopped by adding 250 μL of 1 M Na_2_CO_3_. Activity was calculated in Miller units.

### RNA isolation, cDNA synthesis, and qRT-PCR.

Cells were grown to an OD_600_ of 0.7 and harvested. The pellet was resuspended in 1 mL of RNAprotect Bacteria Reagent (Qiagen, Hilden, Germany) and incubated at 25°C for 10 min. The cells were pelleted and resuspended in 250 μL of 50:10 TE buffer (50 mM Tris, 10 mM EDTA) containing 5 μL of 20% SDS. The cell suspensions were mixed with 300 μL acidic phenol and 250 μL glass beads in screw-cap tubes, then subjected to mechanical disruption in a Bead Beater-16 (Biospec Products, Inc., Bartlesville, OK). Total RNA was isolated using the RNeasy Minikit (Qiagen, Hilden, Germany) and treated with RNase-free DNase I (Qiagen). RNA concentration in the samples was determined using a NanoDrop 2000 spectrophotometer (Thermo Fisher Scientific, USA).

cDNAs were produced from 1 μg of total RNA using the SuperScript IV First-Strand Synthesis System (TaKaRa Bio, Shiga, Japan) according to the manufacturer’s instructions. qRT-PCR was performed in a StepOnePlus real-time PCR system using 2× qPCR MasterMix containing EvaGreen, high ROX (Coregen, Busan, South Korea): one cycle of 95°C for 15 min, followed by 40 cycles of 95°C for 30s, 54°C for 30s, and 72°C for 30s. All results were normalized to the 16S rRNA expression. Fold changes in each sample were represented using the threshold cycle (2^–ΔΔCT^) method.

### Phosphoenolpyruvate-dependent phosphotransferase system assay.

Overnight cultures were incubated in tryptone-vitamin vase (TV) medium (49) with 10 mM glucose fructose or sucrose and harvested at an OD_600_ of 0.5. The cell pellets were washed twice with 0.1 M sodium-potassium phosphate buffer containing 5 mM MgCl_2_ (NaKPO_4_ [pH 7.2]) and resuspended in 0.1 volume of the NaKPO_4_ buffer. Furthermore, 0.05 volume of toluene:acetone (1:9, vol/vol) was added to the sample to establish the permeabilized cell and the sample was vortexed twice for 2 min. The reaction mixtures included 10 μL of the permeabilized cell, 25 μM NAD (NADH), 5 mM NaF, 5 units of lactate dehydrogenase (LDH), 5 mM sugar, and 2.5 mM PEP in 0.1 M NaKPO_4_. NADH oxidation was initiated by adding PEP. The ratio of oxidation of NADH by PEP was measured at 37°C for 30 min at 1-min intervals, with absorbance at 340 nm.

### (p)ppGpp measurement.

Detection of (p)ppGpp accumulation was conducted as described previously (27). Cells were grown in the chemically defined medium FMC (50) containing 25 mM glucose up to an OD_600_ of 0.2 and labeled with [^32^P]orthophosphate for an additional 1 h, with or without 500 ng/mL mupirocin to induce (p)ppGpp synthesis. Following incubation, the cells were harvested and nucleotides were extracted using ice-cold 13 M formic acid, followed by three freeze-thaw cycles in a dry-ice-ethanol bath. Acid extracts obtained by centrifugation were spotted onto a polyethyleneimine-cellulose (PEI)-cellulose plate (Merck, Darmstadt, Germany) to separate phosphorylated nucleotides using thin-layer chromatography. The plates were chromatographed with 1.5 M KH_2_PO_4_ (pH 3.4), air-dried, and exposed to X-ray film at −80°C.

### Statistical analysis.

All graphical data display the means and standard deviations for a minimum of three biological replicates. Student’s *t* test was conducted for statistical comparisons of the data. In all cases, *P < *0.05 was considered significant.
