# Supplementary material for: CcpA and CodY Regulate CRISPR-Cas System of Streptococcus mutans
Source: Microbiol Spectr. 2023 Jun 27;11(4):e01826-23. doi: 10.1128/spectrum.01826-23 (PMC10434267; doi:10.1128/spectrum.01826-23)
Supplement: Supplemental file 1 — Supplemental material. Download spectrum.01826-23-s0001.docx, DOCX file, 2.8 MB [file spectrum.01826-23-s0001.docx]

Supplementary Materials

**CcpA and CodY regulate CRISPR-Cas system of *Streptococcus mutans***

Da-Young Kang^1^, Andy Kim^2^ and Jeong Nam Kim^1,3^*

^1^Department of Integrated Biological Science, College of Natural Sciences, Pusan National University, Busan 46241, Republic of Korea

^2^Department of Chemistry and Biochemistry, Texas Tech University, Lubbock, TX 79409, USA

^3^Department of Microbiology, College of Natural Sciences, Pusan National University, Busan 46241, Republic of Korea

*** Correspondence:**

Department of Microbiology

Pusan National University, College of Natural Sciences

Biology building 402, Busandaehang-ro,

63beon-gil, Geumjeong-gu,

Busan 46241, South Korea

Phone: 82-51-510-2269

Fax: 82-51-514-1778

E-mail: [kimjn@pusan.ac.kr](mailto:kimjn@pusan.ac.kr)

**TABLE S1.** Bacterial strains and plasmids used in this study.

| Strain and plasmid^a^ | Genotype or characteristics | Reference |
| --- | --- | --- |
| Strain |  |  |
| *Streptococcus mutans* |  |  |
| UA159 | Wild-type (WT) |  |
| WT-P_CR1_*_cas_*-*lacZ* | A WT strain carrying *lacZ* gene fusion to CRISPR1-Cas promoter, Km^r^ | This study |
| WT-P_CR2_*_cas_*-*lacZ* | A WT strain carrying *lacZ* gene fusion to CRISPR2-Cas promoter, Km^r^ | This study |
| Δ*ccpA* | *ccpA* gene deletion, NPErm^r^ | (1) |
| Δ*ccpA-*P_CR1_*_cas_*-*lacZ* | *ΔccpA* carrying CRISPR1-Cas promoter fused to *lacZ* gene | This study |
| Δ*ccpA-*P_CR2_*_cas_*-*lacZ* | *ΔccpA* carrying CRISPR2-Cas promoter fused to *lacZ* gene | This study |
| Δ*codY* | *codY* gene deletion | (2) |
| Δ*codY-*P_CR1_*_cas_*-*lacZ* | *ΔcodY* carrying CRISPR1-Cas promoter fused to *lacZ* gene | This study |
| Δ*codY-*P_CR2_*_cas_*-*lacZ* | *ΔcodY* carrying CRISPR2-Cas promoter fused to *lacZ* gene | This study |
| ΔCR1*cas* | Deletion of *cas* genes in CRISPR1 | This study |
| ΔCR1*cas-*P*relA-lacZ* | *Δ*CR1*cas* carrying *relA* promoter fused to *lacZ* gene | This study |
| ΔCR2*cas* | Deletion of *cas* genes in CRISPR2 | This study |
| ΔCR2*cas-*P*relA-lacZ* | *Δ*CR2*cas* carrying *relA* promoter fused to *lacZ* gene | This study |
| ΔCRD*cas* | Deletion of *cas* genes in both CRISPRs | This study |
| ΔCRD*cas-*P*relA*-*lacZ* | *Δ*CRD*cas* carrying *relA* promoter fused to *lacZ* gene | This study |
|  |  |  |
| *Escherichia coli* |  |  |
| His_6_-CcpA | *ccpA* coding region cloned into pQE, Amp^r^ | (1) |
| His_6_-CodY | *codY* coding region cloned into pET-30a, Amp^r^ | (3) |
|  |  |  |
| plasmid |  |  |
| pJL105 | Integration vector of *Streptococcus mutans* UA159, Km^r^, Spec^r^ | (4) |
| pMZ-*lacZ* | Integration vector of *Streptococcus mutans* UA159  *lacZ* gene reporter plasmid, Km^r^, Erm^r^ | (5) |
| P_CR1_*_cas_*-*lacZ* | CRISPR1-Cas promoter fused to *lacZ* gene | This study |
| P_CR2_*_cas_*-*lacZ* | CRISPR2-Cas promoter fused to *lacZ* gene | This study |
| P*relA-lacZ* | *relA* promoter fused to *lacZ* gene | This study |

^a^ CR, CRISPR

**TABLE S2.** Oligonucleotides used in this study.

| Primer name | Nucleotide sequence (5’- 3’) |
| --- | --- |
| Primers for *lacZ* fusion |  |
| Upstream *cas* operon in CR1 FP | TTTTTTGAGCTCACCGATCCGCTGGATAAT |
| Upstream *cas* operon in CR1 RP | TTTTTTGGATCCAACAGCCCAACCAACAG |
| Upstream *cas* operon in CR2 FP | TTTTTTGAGCTCGGGACTTTGTCTTTCTGAAT |
| Upstream *cas* operon in CR2 RP | TTTTTTGGATCCTTACCTCGCTAGTCATTG |
|  |  |
| Primers for quantitative Real-time PCR |  |
| 16s rRNA FP | CTTACCAGGTCTTGACATCC |
| 16s rRNA RP | ACCCAACATCTCACGACAC |
| SMU.1405c FP | ACGGCTATGCGGGTTATATT |
| SMU.1405c RP | CTTAGAAAATCTTCACGCTCAA |
| SMU.1404c FP | ACCCTAGTAATCGAGAAGGAC |
| SMU.1404c RP | ACAGCAGCGTATAACCATAGTC |
| SMU.1403c FP | TACTGCTGAGGAACGCAAA |
| SMU.1403c RP | TGGCTGTATTGGCAGAGTTAT |
| SMU.1402c FP | ACTGGCAAATACCATTACGG |
| SMU.1402c RP | TGACGCCTAAAGCTTTGATTA |
| SMU.1764c FP | AGTCCAACCACAAGGCTTA |
| SMU.1764c RP | TAAGATTCGTCTTCCCAGC |
| SMU.1763c FP | AGAGTGCTTTGGCTTGGT |
| SMU.1763c RP | TTGTTGGATAGGCGAAAGA |
| SMU.1761c FP | AAATGAAAATGCTTCATCAGAC |
| SMU.1761c RP | TGAGAGGTACCTAACCAAGTATGAC |
| SMU.1760c FP | GATGAGTGACGTTTCCATT |
| SMU.1760c RP | AACGTTGTTTCAAGGAATAAATAC |
| SMU.1758c FP | ATTGTTCAGCTGGTAGCACA |
| SMU.1758c RP | CCTCCGTAATTTCAATGACC |
| SMU.1755c FP | GCCAAACGGTTTATCCTG |
| SMU.1755c RP | GTCAACTCAGTATTGACTTCCTCA |
| SMU.1753c FP | CATGTCGCAAAACTCTGTG |
| SMU.1753c RP | CTTGGTCAATGATTGTTGAGAG |
|  |  |
| Primers for mutant construction |  |
| *cas* operon in CR1_up flanking FP | AGCATCAGAATCAGTCCAAG |
| *cas* operon in CR1_up flanking RP | TTTTTTGAGCTCGCAGTATTCCCGCTATC |
| *cas* operon in CR1_down flanking FP | TTTTTTGGATCCGTTTTATTTCTTGAACCC |
| *cas* operon in CR1_down flanking RP | TAATTTACTCGGTCACGGTC |
| *cas* operon in CR2_up flanking FP | CGCTTGCTTTCAGTTATAC |
| *cas* operon in CR2_up flanking RP | TTTTTTGAGCTCTTACCTCGCTAGTCATTG |
| *cas* operon in CR2_down flanking FP | TTTTTTGGATCCGCACATCAAAACCAGAGA |
| *cas* operon in CR2_down flanking RP | TGACGAAACTAACCAATGAG |
| Kanamycin cassette FP | TTTTTTGAGCTCGACATCTAAATCTAGGTACTAAAA |
| Kanamycin cassette RP | TTTTTTGGATCCGCAGATTGCCTTGAATA |
| Spectinomycin cassette FP | TTTTTTGAGCTCCCGTTAGTTGAAGAAGG |
| Spectinomycin cassette RP | TTTTTTGGATCCCTTGCTCAATCAATCAC |
|  |  |
| Primers for EMSAs |  |
| Upstream of *cas* operon in CR1 FP | GATACACTTGAAGATCCATTTGA |
| Upstream of *cas* operon in CR1 RP | /5’-Bio/ TTCGGTGCTTTTTTATTTGC |
| Upstream of *cas* operon in CR2 FP | GCGGATATTCCAAAACTTA |
| Upstream of *cas* operon in CR2 RP | /5’-Bio/ TTACCTCGCTAGTCATTG |
|  |  |
| Primers for DNase I footprinting |  |
| Upstream of *cas* operon in CR1 FP | /5’-6FAM/ GATACACTTGAAGATCCATTTGA |
| Upstream of *cas* operon in CR1 RP | /5’-Bio/ TTCGGTGCTTTTTTATTTGC |
| Upstream of *cas* operon in CR2 FP | /5’-6FAM/ GCGGATATTCCAAAACTTA |
| Upstream of *cas* operon in CR2 RP | /5’-Bio/ TTACCTCGCTAGTCATTG |

(A)

**
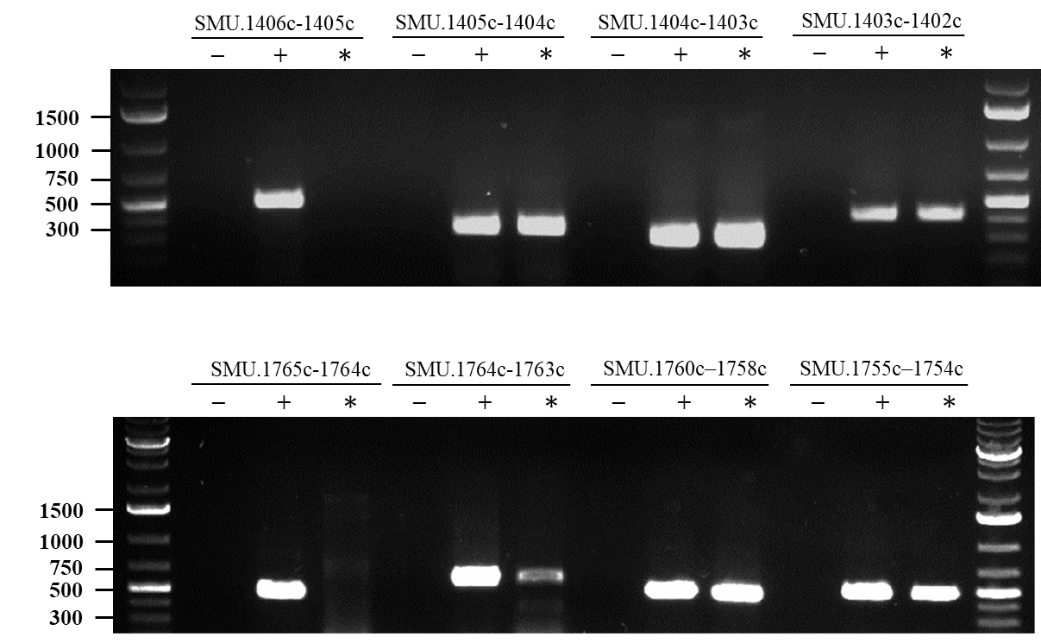
**

(B)

**Figure S1. The results of reverse transcription PCR (RT-PCR).** A pair of specific primers targeting interval regions between *cas* genes were used in reverse transcription and PCR reactions. The PCR products obtained for the interval regions between *cas* genes of CRISPR1-*cas* (A) and CRISPR2-*cas* (B) are shown. Non-template DNA, chromosomal DNA control, and the RT-PCR results are represented by “-”, “+”, and “*”, respectively.


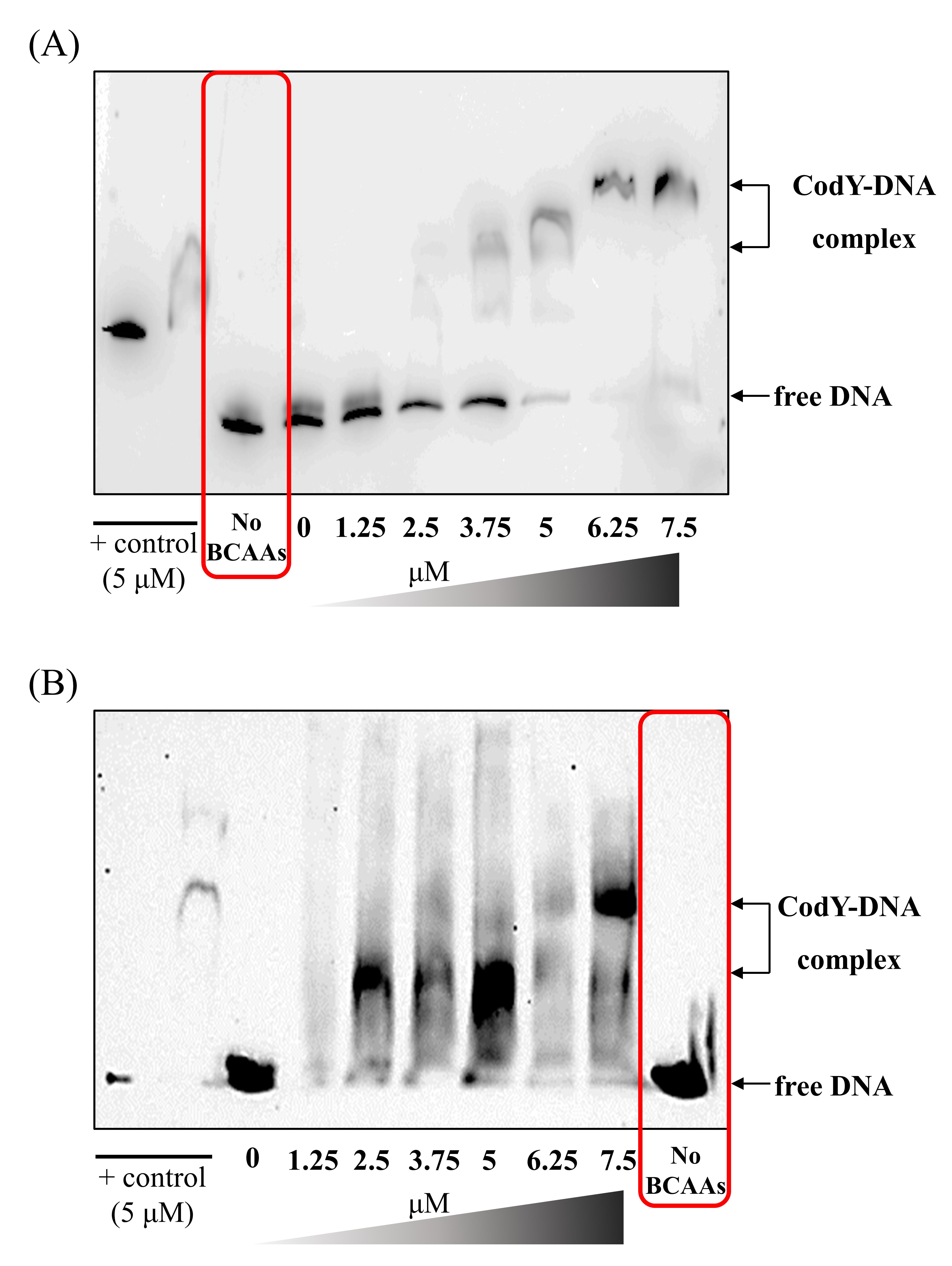


**Figure S2. *In vitro* binding of CodY to the promoter of the *cas* operons.** Biotin-labelled promoter DNA (2.5 ng) was used as a probe and incubated with purified CodY protein (1.25-7.5 μM) at 37°C for 30 min. The direct interaction of CodY with the biotinylated CRISPR1 promoter (A) and the CRISPR2 promoter (B) was examined. The *codY* probe was used as a positive (+) control. Branched-chain amino acids (BCAAs) were added as effector molecules at a final concentration of 10 mM. The arrows indicate the migration of the protein-DNA complex or free (unbound) DNA. The red boxes indicate the absence of a shift in the CodY binding reaction to each promoter without the addition of BCAAs.

**References**

1. Abranches J, Nascimento MM, Zeng L, Browngardt CM, Wen ZT, Rivera MF, Burne RA. 2008. CcpA regulates central metabolism and virulence gene expression in *Streptococcus mutans*. J Bacteriol 190:2340-9.

2. Lemos JA, Nascimento MM, Lin VK, Abranches J, Burne RA. 2008. Global regulation by (p)ppGpp and CodY in *Streptococcus mutans*. J Bacteriol 190:5291-9.

3. Kim JN, Burne RA. 2017. CcpA and CodY Coordinate Acetate Metabolism in *Streptococcus mutans*. Appl Environ Microbiol 83.

4. Kim JN, Ahn SJ, Seaton K, Garrett S, Burne RA. 2012. Transcriptional organization and physiological contributions of the *relQ* operon of *Streptococcus mutans*. J Bacteriol 194:1968-78.

5. Son M, Ahn SJ, Guo Q, Burne RA, Hagen SJ. 2012. Microfluidic study of competence regulation in *Streptococcus mutans*: environmental inputs modulate bimodal and unimodal expression of *comX*. Mol Microbiol 86:258-72.
